# Small cell carcinoma of the ovary hypercalcemic type (SCCOHT): A rare case after in vitro fertilization (IVF)

**DOI:** 10.12669/pjms.331.11634

**Published:** 2017

**Authors:** Ahmed Ghazi, Aqueela Ayaz, Tahira Hamid, Mian Usman Farooq, Nikita Islam

**Affiliations:** 1 Ahmed Ghazi, MBBS, Saudi board. Department of Gyne Oncology, King Abdullah Medical City (KAMC-HC), Makkah, Saudi Arabia; 2 Aqueela Ayaz, MBBS, FCPS. Department of Gyne Oncology, King Abdullah Medical City (KAMC-HC), Makkah, Saudi Arabia; 3 Tahira Hamid, MBBS, FCPS. Department of Laboratory & Blook Bank, King Abdullah Medical City (KAMC-HC), Makkah, Saudi Arabia; 4 Mian Usman Farooq, MBBS, MBA, MSc. Department of Strategic Planning and Institutional Advancement, King Abdullah Medical City (KAMC-HC), Makkah, Saudi Arabia; 5 Nikita Islam, MBBS, MD, MRCOG. Department of Gyne Oncology, King Abdullah Medical City (KAMC-HC), Makkah, Saudi Arabia

**Keywords:** Ovarian cancer, Small cell carcinoma, Hypercalcemia, SCCOHT, In Vitro fertilization

## Abstract

Small cell carcinoma of the ovary, hypercalcemic type (SCCOHT) is a very rare and lethal tumor, mostly affecting young women, with aggressive clinical course. It has a worse prognosis in younger women and most of them died within two years of diagnosis. We are reporting a unique case of SCCOHT in a 35 years old, nulliparous lady with primary infertility in which symptomatic hypercalcemia was a presenting feature of her cancer. She was completely healthy before third cycle of IVF. Within two months of her third IVF cycle, she developed SCCOHT with a very rapid and aggressive course of disease and fatal outcome. Patient died within one month of her first symptom presentation (3 months after IVF cycle).

## INTRODUCTION

Small cell carcinoma of the ovary, hypercalcemic type (SCCOHT) is exceedingly rare tumor with very aggressive behavior and strikingly poor prognosis. It predominantly affects young women with average age 23-24 years (range 10-49 years).^1^ Two third of these cases are associated with hypercalcemia. However, symptoms of hypercalcemia, e.g., polyuria, polydipsia, anorexia, nausea, vomiting, constipation, muscle weakness, bone pain, decreased concentration, confusion, fatigue and coma was reported in only 2.5% of these patients.^2^

The mechanism of development of hypercalcemia associated with this tumor is unclear. It has been postulated that parathyroid hormone or parathyroid hormone-related protein (PTHrp), or a tumor induced calcium release by non-tumor related tissues may play a vital role.^3^

Almost 300 cases have been reported in literature.^4^ SCCOHT at first presentation is generally in FIGO stage III. The 1- year survival is 50%, and overall 5-year survival rate is approximately 10%.^1^

To the best of our knowledge, SCCOHT in a nulliparous woman with primary infertility after invitro fertilization has not been published in literature yet.

### Ethical issues

The institutional review board of King Abdullah Medical City, Makkah, Saudi Arabia granted us approval to report this case.

## CASE REPORT

We present the case of a Thirty five year old nulliparous Saudi teacher married, for 11 years. She was transferred from a private hospital to King Abdullah Medical city a tertiary care Oncology Centre, Makkah as a case of primary infertility with multiple abdominal masses. She presented with progressive enlargement of abdomen associated with abdominal pain, nausea, constipation, anorexia and insomnia from last one month with worsening of symptoms from last three days. She had weight loss 5kg over last one month. She was fatigued and lethargic for last one week with difficulty in walking for the last three days. She had respiratory difficulty for one day. She was in a good state of health upto three months back, when she underwent a third IVF cycle after two failed IVF attempts, three years and six months back, respectively. Third IVF attempt was also unsuccessful with uneventful post IVF period for almost 2 months. Other than IVF treatment there was no significant past medical, surgical or family history. She had no known allergies.

On examination, she was conscious, oriented, responding well with Glasgow coma scale 15/15 and body mass index 26. Her blood pressure was 120/60 mm of Hg, pulse-122/min, temp 36.2°C, respiratory rate 28/minute, oxygen saturation 94% on room air. She was pale, jaundiced with bilateral lower limb edema. Abdomen was enlarged and tense, with generalized tenderness all over the abdomen. There was a diffuse solid mass occupying whole upper and lower abdomen along with hepatomegaly but no shifting dullness or fluid thrill. Bimanual examination revealed a very short anteriorly placed cervix, with pinpoint os. Uterus was not felt separately. Pouch of Douglas and both fornices was full with mass. Per rectal exam showed collapsed rectum with external compression.

Prompt work up was started. Laboratory tests showed hemoglobin 9.9g/dl with normal platelet count but abnormal coagulation profile, i.e., INR (3.2). A white blood cell count was 29.5X10^9^/L. C-reactive protein 42 mg/dl and procalcitonin were 11ng/dl. Deranged liver function tests and renal function tests, i.e., AST (218 U/L), ALT (144 U/L), conjugated bilirubin (2.95 mg/dl), total bilirubin (3.58 mg/dl), GGT (362), BUN (34), serum creatinine (2.1 mg/dl) and uric acid (9.9 mg/dl). Corrected calcium was raised to 17.58 mg/dl with raised phosphorous (6.5 mg/dl) but potassium, magnesium was normal with low Na (123 mmol/L) and chloride (87 mmol/L). Thyroid function test and parathyroid hormone were normal. Hepatitis and HIV serology was negative. Septic work up including blood, urine, sputum culture sensitivity (C/S), MRSA, Gram staining, AFB and rectal swab for pseudomonas, VRE, CRE all were negative. Tumor markers showed raised LDH (1728 U/ml) and CA 125 (77U/ml) but other markers CA 19.9, CA 15.3, CEA, AFP, hCG were normal. Arterial blood gases revealed metabolic acidosis with raised lactate. Computed tomographic scan of chest, abdomen and pelvis revealed bilateral pulmonary nodule, significantly enlarged liver with multiple focal lesion. There was an ovarian mass of 13.5×17.9×11.9cm inseparable from the uterus, right ureter and urinary bladder. There were mesenteric and omental nodules and widely scattered peritoneal lesions with abdominopelvic lymphadenopathy.

**Fig. 1:**
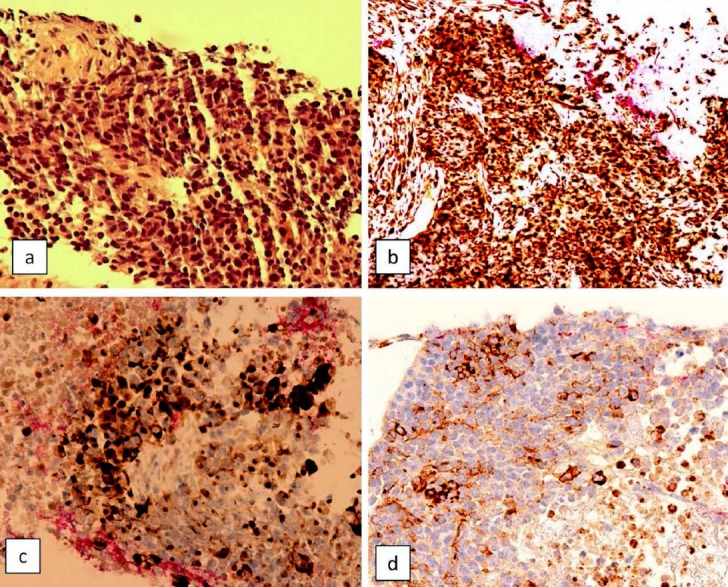
a. Hematoxylin and eosion stain (40x): Small cell carcinoma showing sheets of small rounded cells with hyperchromatic nuclei and scanty cytoplasm. b. Vimentin immunostain (40x): tumor cells are strongly positive for vimentin. c. CK AE1/AE3 immunostain (40x): tumor cells are positive for CK AE1/AE3. d. Synaptophysin immunostain (40x): tumor cells are focally positive for Synaptophysin.

The patient was seen by a multidisciplinary team including internal medicine, nephrology, urology, endocrinology and intensivist and transferred to Intensive care unit with diagnosis of metabolic acidosis with raised lactate and hypercalcemia with oliguria. Patient received supportive treatment, broad spectrum antibiotic and calcitonin. Ultrasound guided tru-cut biopsy of pelvic mass was done. The patient’s condition worsened very rapidly. She became confused, desaturated, hypotensive, febrile and anuric, with GCS 8/15. Patient was intubated, received inotropic support and continuous renal replacement therapy was started. Histopathology of pelvic mass came as small cell carcinoma of ovary, hypercalcemic type (Fig. 1). A family meeting was conducted and patient declared as DNR (Do not resuscitate). She passed away four days after admission, within one month of her first symptom presentation.

## DISCUSSION

SCCOHT is a very lethal tumor that typically affects young women, and most of them died within a year of diagnosis^5^ Scully briefly mentioned monograph of this tumor in 1979 but Dickersin *et al.*, in 1982 first reported eleven cases of a small cell ovarian cancer associated with hypercalcemia and compared with three similar cases in literature^6^,^7^ There are two different varieties of small cell carcinoma of the ovary, one includes a large cell component (large cell variant of SCCO) which is more uncommon and less known and the other is similar to small cell carcinoma of lung.

Our case is different from other reported cases. Our patient was younger (35-year-old) nulliparous with primary infertility and developed SCCOHT after IVF cycle and died three months after IVF cycle. To evaluate the cancer risk among women treated for infertility is very complex. Many factors lead to the development of cancer in these patients. On one hand, nulliparity and infertility are established risk factor for ovarian cancer. On the other hand, infertility treatment like ovulation induction is linked to higher incidence of ovarian cancer. In recent years, the relationship between infertility, fertility treatments, and the risk of gynecological malignancies has aroused much interest, mainly for breast, uterus and ovarian cancer.^8^,^9^

In past decades many case-control studies failed to show a significant relationship between fertility drugs use and ovarian cancer risk.^10^ However some recent studies have concluded that ovarian stimulation for IVF may increase the risk of ovarian malignancies, especially borderline ovarian tumors or epithelial ovarian cancer.^11^

To the best of our knowledge our case is the first reported case of SCCOHT which developed after two months of ovarian stimulation for IVF and had a grave prognosis with the patient passing away within one-month after first symptom and three months after ovarian stimulation for IVF.

This raises the query-Is this by chance? Does ovarian stimulation for IVF trigger some gene mutation or an underlying microscopic pathology in ovarian cells leading to development of such rapid and aggressive tumor with mortal prognosis in a very short time period?

Knowledge about the extent of risk associated with ovarian stimulation, specifically in relation to gynecological malignancies is important for women considering IVF treatment and needs to be addressed by multicenteric double blind randomized placebo controlled trial on large number of subjects.
